# Contemporary Multimodal Management of Cutaneous Malignancies: Surgery, Radiotherapy, Systemic Therapy, Molecular Targeting and Immunotherapy

**DOI:** 10.3390/cancers18183029

**Published:** 2026-09-18

**Authors:** Dariusz Kowalczyk, Jolanta Jaworek, Andrzej Ciborek, Pawel Antoni Kolodziejski, Ewa Pruszyńska-Oszmałek, Hanna Krauss

**Affiliations:** 1 Faculty of Medicine and Health Sciences, University of Kalisz, 62-800 Kalisz, Poland; d.kowalczyk@uniwersytetkaliski.edu.pl (D.K.); a.ciborek@uniwersytetkaliski.edu.pl (A.C.); 2 Department of Medicine and Health Care, Faculty of Medicine, University of Applied Sciences in Tarnow (Akademia Tarnowska), ul. Mickiewicza 8, 33-100 Tarnow, Poland; jola_jaworek@poczta.onet.pl; 3 Department of Animal Physiology and Biochemistry, Poznan University of Life Sciences, 60-637 Poznan, Poland; pawel.kolodziejski@up.poznan.pl (P.A.K.); ewa.pruszynska@up.poznan.pl (E.P.-O.)

**Keywords:** skin cancer, melanoma, cutaneous squamous-cell carcinoma, basal-cell carcinoma, Merkel-cell carcinoma, immune checkpoint inhibitors, neoadjuvant immunotherapy, BRAF/MEK inhibitors, Hedgehog-pathway inhibitors, cemiplimab

## Abstract

Background: The therapeutic management of cutaneous malignancies—cutaneous melanoma, cutaneous squamous-cell carcinoma (cSCC), basal-cell carcinoma (BCC) and Merkel-cell carcinoma (MCC)—has shifted toward multimodal, stage-adapted treatment integrating surgery, radiotherapy, molecularly targeted therapy and immune checkpoint inhibition. Methods: We conducted a structured narrative review with a structured literature search of PubMed/MEDLINE, Scopus and Web of Science (January 2015–6 September 2026), prioritising current clinical practice guidelines, randomised controlled trials and phase II–III studies; landmark older trials were included separately. Synthesis: Surgery with adequate histological margins, including Mohs micrographic surgery where appropriate, remains definitive for localised disease. In resectable macroscopic stage III melanoma, neoadjuvant ipilimumab plus nivolumab with response-driven postoperative management improved event-free survival versus adjuvant nivolumab in NADINA. SWOG S1801 independently demonstrated an event-free-survival advantage for perioperative pembrolizumab over adjuvant-only pembrolizumab in resectable stage III–IV melanoma. In advanced melanoma, dual immune-checkpoint strategies and BRAF/MEK inhibition require patient-specific sequencing; in the European Union, nivolumab plus relatlimab is authorised for first-line advanced melanoma with tumour-cell PD-L1 expression <1%. In high-risk cSCC after surgery and radiotherapy, adjuvant cemiplimab improved disease-free survival. In advanced BCC, Hedgehog-pathway inhibitors remain the principal first systemic option, with anti-PD-1 therapy after HHI failure or intolerance. Clinical implications: Molecular testing should be indication-driven: BRAF V600 is a validated treatment-selecting biomarker, while broader NGS profiling (including NRAS, KIT, NF1, TERT and co-occurring alterations), PD-L1, tumour mutational burden and circulating tumour DNA have context-dependent, supportive or investigational roles. Conclusions: The major unresolved issues are optimal sequencing, response-adapted perioperative treatment, resistance, biomarker validation and treatment of transplant, frail and other under-represented populations.

## Simple Summary

Cutaneous malignancies include melanoma, cutaneous squamous-cell carcinoma, basal-cell carcinoma and Merkel-cell carcinoma. Their management has changed rapidly in recent years owing to the increasing use of immune checkpoint inhibitors, targeted therapies and perioperative systemic treatment. This review summarizes the current multimodal treatment landscape, including surgery, radiotherapy, immunotherapy, molecularly targeted therapy and selected reconstructive strategies. Particular attention is given to recent practice-changing evidence, including neoadjuvant immunotherapy in resectable stage III melanoma, adjuvant cemiplimab in high-risk cutaneous squamous-cell carcinoma, anti-LAG-3 therapy in advanced melanoma and Hedgehog-pathway inhibition in advanced basal-cell carcinoma. The review also discusses predictive biomarkers, treatment sequencing, resistance mechanisms and special populations such as transplant recipients. Overall, treatment selection is increasingly determined by tumour type, stage, resectability and validated biomarkers, while several important questions concerning optimal sequencing, long-term outcomes and under-represented patient groups remain unresolved.

## 1. Introduction

The treatment of cutaneous malignancies has changed substantially over the past fifteen years. The approval of vemurafenib for BRAF V600E-mutated melanoma [1] and the survival benefit shown with ipilimumab [2], followed by the anti-PD-1 antibodies pembrolizumab and nivolumab [3,4], raised the median survival in advanced melanoma from under one year to durable five-year remission in a substantial proportion of patients [5,6]. More recent gains have reached the non-melanoma skin cancers: Hedgehog-pathway inhibitors for advanced basal-cell carcinoma (BCC) [7,8] and anti-PD-1 therapy for advanced cutaneous squamous-cell carcinoma (cSCC) [9,10] and Merkel-cell carcinoma (MCC) [11,12].

Several practice-changing trials reported between 2022 and 2026 motivate this review. In resectable macroscopic stage III melanoma, the phase 3 NADINA trial showed superior event-free survival with neoadjuvant ipilimumab plus nivolumab followed by response-driven postoperative management compared with adjuvant nivolumab [13,14]. SWOG S1801 likewise demonstrated that giving pembrolizumab before and after surgery improved event-free survival compared with adjuvant-only pembrolizumab in resectable stage III–IV melanoma [15]. In advanced melanoma, nivolumab plus relatlimab is a first-line dual-checkpoint option; importantly, the current European Medicines Agency indication is restricted to adults and adolescents aged ≥12 years with unresectable or metastatic melanoma and tumour-cell PD-L1 expression <1% [16]. In high-risk cSCC after surgery and radiotherapy, adjuvant cemiplimab became the first systemic therapy with a randomised disease-free-survival benefit [17]. These advances coexist with persistent therapeutic challenges, including acquired resistance to MAPK-pathway inhibition [18,19,20], immune-related toxicity [21,22], and the need to translate molecular information into multidisciplinary treatment decisions.

This review is organised primarily by tumour type because therapeutic selection, sequencing and molecular testing differ fundamentally among melanoma, keratinocyte carcinomas (cSCC and BCC), and MCC. Within each tumour, the synthesis integrates local and surgical treatment, radiotherapy, perioperative therapy, systemic treatment of advanced disease, predictive and investigational biomarkers, resistance, and evidence gaps. Particular emphasis is placed on how resectability, pathological response, molecular profile, comorbidity, transplant status and anticipated toxicity are incorporated into multidisciplinary decision-making.

## 2. Materials and Methods

This work is a narrative review supported by a purposive, structured literature search; it is not presented as a systematic review and was not conducted under PRISMA. The search was designed to identify and critically integrate clinically relevant evidence across biologically and methodologically heterogeneous cutaneous malignancies rather than to provide exhaustive study identification or quantitative synthesis. PubMed/MEDLINE, Scopus and Web of Science were searched for English-language records published from January 2015 to 6 September 2026. Reference lists, regulatory documents and current clinical practice guidelines were also examined. Landmark studies published before 2015 were retained when they established a therapeutic mechanism, registration indication or practice-changing standard. The search was updated for this revision to capture regulatory changes, long-term trial updates and peer-reviewed evidence published in 2026.

Search concepts combined each tumour type (melanoma, cutaneous squamous-cell carcinoma, basal-cell carcinoma and Merkel-cell carcinoma) with intervention and biomarker terms, including immune checkpoint inhibitors, PD-1, PD-L1, CTLA-4, LAG-3, BRAF, MEK, NRAS, KIT, NF1, TERT, next-generation sequencing, circulating tumour DNA, tumour mutational burden, Hedgehog inhibitors, neoadjuvant therapy, adjuvant therapy and radiotherapy. Boolean combinations were adapted to the indexing and syntax of each database. Evidence was prioritised in the following order: current clinical practice guidelines; randomised controlled trials; phase II–III studies; prospective cohorts and meta-analyses; and retrospective or real-world studies addressing questions or populations not adequately represented in pivotal trials. Candidate records were reviewed by two authors, and disagreements about relevance were resolved by discussion. Regulatory status, study design, direct versus indirect comparisons, consistency with the wider evidence base and major limitations were considered during narrative synthesis. Because the purpose was interpretive synthesis rather than exhaustive effect estimation, no formal risk-of-bias instrument, certainty-grading framework or meta-analysis was applied.

## 3. Principles of Risk Stratification and Multidisciplinary Decision-Making

**Figure 1:**
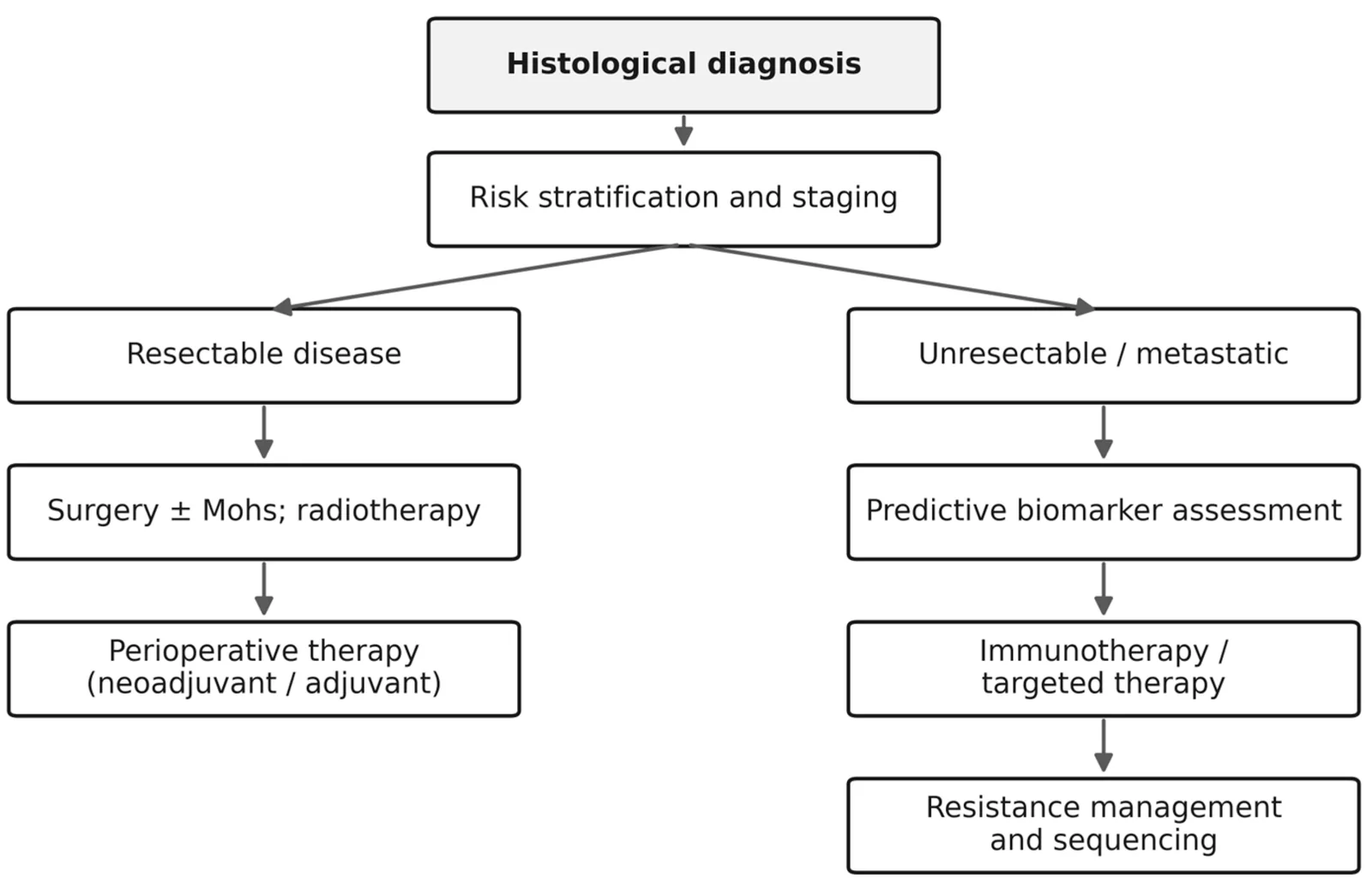
Clinical decision framework for cutaneous malignancies, from histological diagnosis through risk stratification to resectability-driven local, perioperative and systemic treatment. Original figure created by the authors; no third-party figure has been reproduced or adapted. The framework is based on current clinical practice guidelines [23,24,26,39,40].

Across all cutaneous malignancies, management begins with histological diagnosis, accurate staging and structured risk assessment, which together determine resectability and the role of systemic therapy. For melanoma, Breslow thickness, ulceration, mitotic activity, sentinel lymph-node status and disease burden inform prognosis and treatment intensity [23,24,25]. In stage III–IV disease, actionable mutation testing is recommended; BRAF V600 testing is mandatory, while NRAS and KIT testing and broader NGS profiling can be considered in selected settings [25]. For cSCC, high-risk features include substantial nodal disease, extranodal extension, perineural invasion, bone invasion and locally recurrent tumour with additional risk features [17,26]. For BCC, the clinically important distinction is between lesions amenable to local therapy and difficult-to-treat locally advanced disease; metastatic BCC is uncommon. Multidisciplinary decisions should integrate resectability, expected functional and cosmetic morbidity, radiotherapy feasibility, pathological-response information after neoadjuvant therapy, actionable molecular alterations, prior treatment, performance status, comorbidity, transplant or autoimmune status, anticipated immune toxicity and patient preferences. Molecular findings should therefore be interpreted as one component of a tumour board decision rather than as isolated laboratory results. The current treatment landscape is summarised in Table 1. The overall resectability-driven decision pathway is shown in Figure 1.

## 4. Cutaneous Melanoma

### 4.1. Local and Surgical Treatment

Wide local excision with stage-appropriate margins remains the definitive treatment of localised melanoma, with sentinel lymph-node biopsy used for nodal staging and prognostication [23]. Surgery alone is curative in most thin, node-negative tumours; the role of systemic therapy increases with stage and nodal burden.

### 4.2. Adjuvant Therapy

Adjuvant anti-PD-1 therapy is established not only for resected stage III disease but also for completely resected stage IIB/IIC melanoma. KEYNOTE-716 demonstrated improved recurrence-free and distant-metastasis-free survival with pembrolizumab versus placebo in stage IIB/IIC disease [27], and CheckMate 76K demonstrated a significant recurrence-free-survival benefit with nivolumab [28]. Pembrolizumab and nivolumab are authorised in these settings in both the United States and European Union [27,28,42,43]. In stage III disease, pembrolizumab and nivolumab remain established adjuvant options [44,45], while dabrafenib plus trametinib is an evidence-based alternative for resected BRAF V600-mutated stage III melanoma [46]. For stage IIIA and other lower-burden stage III presentations, treatment should be discussed rather than applied mechanically; recurrence risk, sentinel-node tumour burden, primary-tumour features including ulceration and mitotic activity, toxicity, and patient preference should inform the risk–benefit assessment [25]. These considerations are important because adjuvant therapy can reduce recurrence risk while exposing potentially cured patients to immune-related or targeted-therapy toxicity.

### 4.3. Neoadjuvant Therapy

Neoadjuvant immunotherapy is a major recent change in melanoma management. In the phase 3 NADINA trial (*n* = 423), two cycles of neoadjuvant ipilimumab plus nivolumab followed by surgery and pathological-response-adapted postoperative management were compared with surgery followed by adjuvant nivolumab in resectable macroscopic stage III melanoma. At the initial analysis, the estimated 12-month event-free survival was 83.7% versus 57.2% (HR 0.32), and a major pathological response was observed in 59.0% of neoadjuvant-treated patients [13]; the 2025 update maintained the EFS advantage and explored interferon-γ signature, tumour mutational burden and PD-L1 as candidate markers [14]. SWOG S1801 provides independent randomised support for the timing principle: perioperative pembrolizumab (three preoperative doses followed by surgery and postoperative pembrolizumab) improved 2-year event-free survival to 72% versus 49% with adjuvant-only pembrolizumab in resectable stage IIIB–IV melanoma [15]. These studies should not be conflated with a universal regulatory label; as of 6 September 2026, EMA product information for pembrolizumab lists advanced and adjuvant melanoma indications but not a dedicated neoadjuvant melanoma indication [42]. The practical value of response-driven treatment is accompanied by unresolved questions. Pathological complete or major response may identify patients in whom postoperative systemic exposure can potentially be reduced, whereas non-responders may require escalation or alternative therapy; however, the optimal action after each response category is not fully standardised outside protocol-defined pathways. Implementation therefore depends on appropriate patient selection, timely surgery, experienced pathological assessment of treatment response, access to multidisciplinary review and avoidance of both undertreatment and unnecessary postoperative toxicity.

### 4.4. Advanced Disease: Immunotherapy

The biological rationale for checkpoint blockade is well established [47,48,49]. For advanced or metastatic melanoma, anti-PD-1 monotherapy and dual checkpoint blockade are standard systemic options. Final 10-year CheckMate 067 results showed a median overall survival of 71.9 months with nivolumab plus ipilimumab, 36.9 months with nivolumab and 19.9 months with ipilimumab, confirming the durable long-term benefit of PD-1-based treatment [35]. RELATIVITY-047 established nivolumab plus relatlimab as an effective dual-checkpoint regimen versus nivolumab, with improved progression-free survival and a lower frequency of severe treatment-related toxicity than historically observed with an ipilimumab-containing dual blockade [29,50]. In the European Union, however, nivolumab plus relatlimab is authorised in first-line advanced melanoma only when tumour-cell PD-L1 expression is <1% [16]; this regulatory selection rule should not be interpreted as proof that PD-L1 is a generally validated predictive biomarker across melanoma immunotherapy regimens. There is no randomised head-to-head trial comparing nivolumab plus relatlimab with nivolumab plus ipilimumab. An adjusted indirect patient-level comparison suggested broadly similar efficacy with fewer grade 3–4 treatment-related adverse events with nivolumab plus relatlimab, but the non-randomised cross-trial nature of that comparison requires caution [36]. Choice between dual-ICI regimens should therefore consider disease burden, BRAF status, CNS involvement, need for rapid disease control, comorbidity and the treating team’s expertise in anticipating, recognising and managing immune-related adverse events, which has evolved substantially since the earliest checkpoint-inhibitor trials. Key pivotal systemic-therapy trials across cutaneous malignancies are summarised in Table 2.

### 4.5. Advanced Disease: Targeted Therapy

For BRAF V600-mutated melanoma, combined BRAF and MEK inhibition (dabrafenib plus trametinib, vemurafenib plus cobimetinib, encorafenib plus binimetinib) targets the MAPK pathway at sequential nodes and produces rapid, high response rates [30,31,32,33,34]. Triplet strategies that add checkpoint inhibition have produced mixed results. IMspire150 showed a progression-free-survival benefit with atezolizumab plus vemurafenib and cobimetinib and supported US regulatory approval of that triplet [52,53], whereas other triplet programmes have not consistently met primary endpoints or changed routine sequencing. Accordingly, triplet therapy should not be presented as a universally preferred strategy; toxicity, treatment discontinuation and the absence of a clear long-term overall-survival advantage over contemporary sequencing strategies remain important limitations. Long-term OS may be a more clinically discriminating endpoint than early response rate in this setting.

### 4.6. Resistance and Treatment Sequencing

Acquired resistance to BRAF/MEK inhibition, typically through MAPK-pathway reactivation, commonly develops during treatment [18,19,20]. The optimal sequence of immunotherapy and targeted therapy in BRAF V600-mutated advanced melanoma has been addressed prospectively. DREAMseq showed superior 2-year overall survival when nivolumab plus ipilimumab preceded dabrafenib plus trametinib rather than the reverse sequence [54]. SECOMBIT and complementary real-world data further suggest that an immunotherapy-first strategy may delay or reduce the development of brain metastases in patients without baseline CNS disease [55,56]. This potential CNS benefit is particularly relevant when baseline clinical features imply a high future risk of brain metastasis, but it should be interpreted as supportive rather than as a stand-alone treatment-selection biomarker. Conversely, rapidly progressive, symptomatic or very high-burden disease may still justify initial BRAF/MEK inhibition because of the need for rapid tumour reduction. Sequencing should therefore be individualised rather than reduced to a single universal algorithm.

## 5. Cutaneous Squamous-Cell Carcinoma

### 5.1. Local Treatment and Radiotherapy

Surgical excision with adequate histological margins, including Mohs micrographic surgery for selected high-risk and anatomically sensitive lesions, is the definitive treatment for localised cSCC. Prospective Mohs series demonstrate low recurrence rates, although not below 1% across all primary cSCC populations [26,57]. Postoperative radiotherapy should be actively evaluated within multidisciplinary review after resection of high-risk cSCC, with guideline-supported indications including extensive perineural involvement, unresectable or persistently positive margins and substantial nodal disease [26]. The decision should account for anatomical site, margin status, nodal burden, functional morbidity and prior irradiation rather than being applied uniformly to every postoperative patient.

### 5.2. Adjuvant Therapy

The phase 3 C-POST trial provided randomised evidence supporting adjuvant cemiplimab for high-risk cSCC. Patients (*n* = 415) with local or regional disease after surgical resection and postoperative radiotherapy, at high risk of recurrence owing to nodal features (extracapsular extension with a node ≥20 mm or at least three involved nodes) or non-nodal features (in-transit metastases, T4 lesion with bone invasion, perineural invasion, or locally recurrent tumour with an additional risk feature), were randomised to adjuvant cemiplimab or placebo. At a median follow-up of 24 months, disease-free survival events occurred in 24 versus 65 patients (HR 0.32, 95% CI 0.20–0.51; *p* < 0.0001), corresponding to a 68% reduction in the risk of recurrence or death; the estimated 24-month disease-free survival was 87.1% versus 64.1%, with reductions in both locoregional (HR 0.20) and distant recurrence (HR 0.35). Grade ≥3 adverse events occurred in 23.9% versus 14.2%, and discontinuation due to adverse events occurred in 9.8% versus 1.5% [17]. On the basis of these data, the US Food and Drug Administration approved cemiplimab as an adjuvant therapy for high-risk cSCC in October 2025 [58]. Overall survival did not differ at the first analysis, in part reflecting a crossover design permitting cemiplimab at relapse. Its place relative to surveillance and treatment at recurrence should therefore be individualised and interpreted in the context of jurisdiction-specific regulatory status.

### 5.3. Advanced Disease and Neoadjuvant Strategies

For locally advanced or metastatic cSCC not amenable to curative surgery or radiotherapy, anti-PD-1 therapy is standard; cemiplimab and pembrolizumab produce clinically meaningful and often durable responses [9,10]. Cosibelimab is another approved option in metastatic or locally advanced disease [37]. EMPOWER-CSCC-1 established the activity of cemiplimab, and recent real-world data suggest that response may vary by anatomical site; one 2026 cohort reported a higher response rate in head-and-neck primaries than in non-head-and-neck disease [51]. Because these site-specific observations are retrospective and not validated as predictive criteria, they should inform discussion and hypothesis generation rather than determine treatment eligibility. Neoadjuvant cemiplimab has produced high pathological-response rates in resectable stage II–IV (M0) cSCC [59], but its precise relationship to established surgery-based pathways remains under active definition.

### 5.4. Special Consideration: Organ-Transplant Recipients

cSCC is markedly more common and more aggressive in solid-organ transplant recipients. These patients were excluded from pivotal cemiplimab studies, including the C-POST adjuvant trial, limiting direct generalisability of registration evidence [9,17,60]. Published prospective checkpoint-inhibitor experience is still concentrated predominantly in kidney-transplant recipients. A phase I study of cemiplimab in kidney-transplant recipients with advanced cSCC demonstrated that treatment can be feasible under a carefully designed immunosuppressive strategy, but these findings cannot be automatically extrapolated to heart, lung, liver or other grafts [61]. Across solid-organ transplant recipients, PD-1 blockade may precipitate allograft rejection, creating a direct conflict between antitumour efficacy and graft preservation. Treatment therefore requires individualised multidisciplinary discussion involving oncology, transplant medicine and the patient, with explicit consideration of immunosuppression modification, rejection risk, alternative local therapies and the consequences of graft failure.

## 6. Basal-Cell Carcinoma

### 6.1. Local Treatment

The majority of BCCs are cured by surgical excision or, for high-risk and facially located lesions, Mohs micrographic surgery [62,63]. Radiotherapy is an option for patients unsuitable for surgery, and superficial modalities (topical agents, photodynamic therapy and cryotherapy) are appropriate for selected low-risk superficial tumours [64,65,66].

### 6.2. Advanced Disease: Hedgehog-Pathway Inhibitors

Metastatic BCC is rare; in routine practice, the more common systemic-treatment scenario is difficult-to-treat, locally advanced or recurrent unresectable BCC. Hedgehog-pathway inhibitors are the principal first systemic option where surgery or radiotherapy cannot achieve acceptable disease control [39]. Hedgehog-pathway dysregulation, usually involving PTCH1 or SMO, is central to BCC biology [8,39]. Vismodegib and sonidegib have clinically meaningful activity [7,8], but cumulative adverse effects—including muscle spasms, dysgeusia, alopecia, decreased appetite and weight loss—can undermine long-term adherence and lead to treatment interruption or discontinuation. These tolerability limitations are clinically important when deciding whether to continue, interrupt, rechallenge or transition to immunotherapy.

### 6.3. Advanced Disease: Immunotherapy After Hedgehog-Inhibitor Therapy

Anti-PD-1 therapy is established for advanced BCC after failure of, contraindication to, or intolerance of Hedgehog-pathway inhibitors. Cemiplimab provides a defined second-line systemic option in this setting [38,39]. Selection among surgery, radiotherapy, Hedgehog inhibition and immunotherapy depends on resectability, prior therapy and patient factors.

## 7. Merkel-Cell Carcinoma

Merkel-cell carcinoma is a rare, aggressive neuroendocrine skin cancer associated with Merkel-cell polyomavirus, ultraviolet exposure and immunosuppression [40]. Localised disease is treated with surgery and, frequently, adjuvant radiotherapy according to dedicated guideline recommendations [40]. For advanced disease, anti-PD-(L)1 therapy has replaced cytotoxic chemotherapy as the preferred systemic approach; avelumab and pembrolizumab produce high and durable objective response rates [11,12], and retifanlimab is an additional evidence-based option for recurrent locally advanced or metastatic MCC, with a US regulatory indication [41,67]. Perioperative immunotherapy is under active investigation, and management after checkpoint-inhibitor resistance remains an unmet need.

## 8. Rare Cutaneous Malignancies

Rare cutaneous malignancies—including dermatofibrosarcoma protuberans (DFSP), sebaceous carcinoma, microcystic adnexal carcinoma and other cutaneous adnexal carcinomas—are generally managed by complete surgical excision with comprehensive margin assessment; Mohs micrographic surgery is appropriate for selected tumours, particularly when tissue preservation and complete peripheral and deep margin control are important [68,69]. The evidence base for systemic treatment of adnexal carcinomas remains limited and is derived mainly from retrospective series and disease-specific reviews [69]. DFSP is characterised in most cases by a COL1A1::PDGFB fusion. Imatinib may be considered for fusion-positive, unresectable, recurrent or metastatic disease and in selected neoadjuvant settings after multidisciplinary assessment [68]. Owing to the rarity and biological heterogeneity of these entities, management should be centralised where possible and guided by tumour-specific pathology and multidisciplinary review.

## 9. Radiotherapy and Combined-Modality Treatment

Radiotherapy has definitive, adjuvant and palliative roles across cutaneous malignancies: as primary treatment for patients unsuitable for surgery, as postoperative therapy in selected high-risk cSCC and MCC, and for symptom control in advanced disease [26,39,40]. Combinations of radiotherapy with checkpoint inhibition are biologically plausible and under clinical investigation; however, their optimal scheduling and incremental clinical benefit remain incompletely defined, and routine use outside established indications or clinical trials is not yet supported. Radiotherapy may influence tertiary lymphoid structures (TLSs) bidirectionally. Immunogenic tumour-cell death can increase antigen release and presentation, while cGAS-STING-dependent type I interferon signalling, inflammatory chemokines, vascular remodelling and lymphocyte recruitment may support TLS formation or functional maturation and thereby enhance local and systemic antitumour immunity. Conversely, high-dose or large-volume irradiation and irradiation of tumour-draining lymph nodes can deplete radiosensitive lymphocytes and damage stromal organiser cells, high endothelial venules and lymphatic trafficking, disrupting established TLSs or preventing their maturation. The net effect is therefore likely to depend on dose, fractionation, irradiated volume, nodal exposure and the timing of radiotherapy relative to immune checkpoint inhibition. TLS-preserving or TLS-promoting radiation strategies are biologically compelling but remain investigational and require prospective validation before they can guide routine sequencing or treatment planning [70].

## 10. Predictive Biomarkers and Molecular Profiling

**Figure 2:**
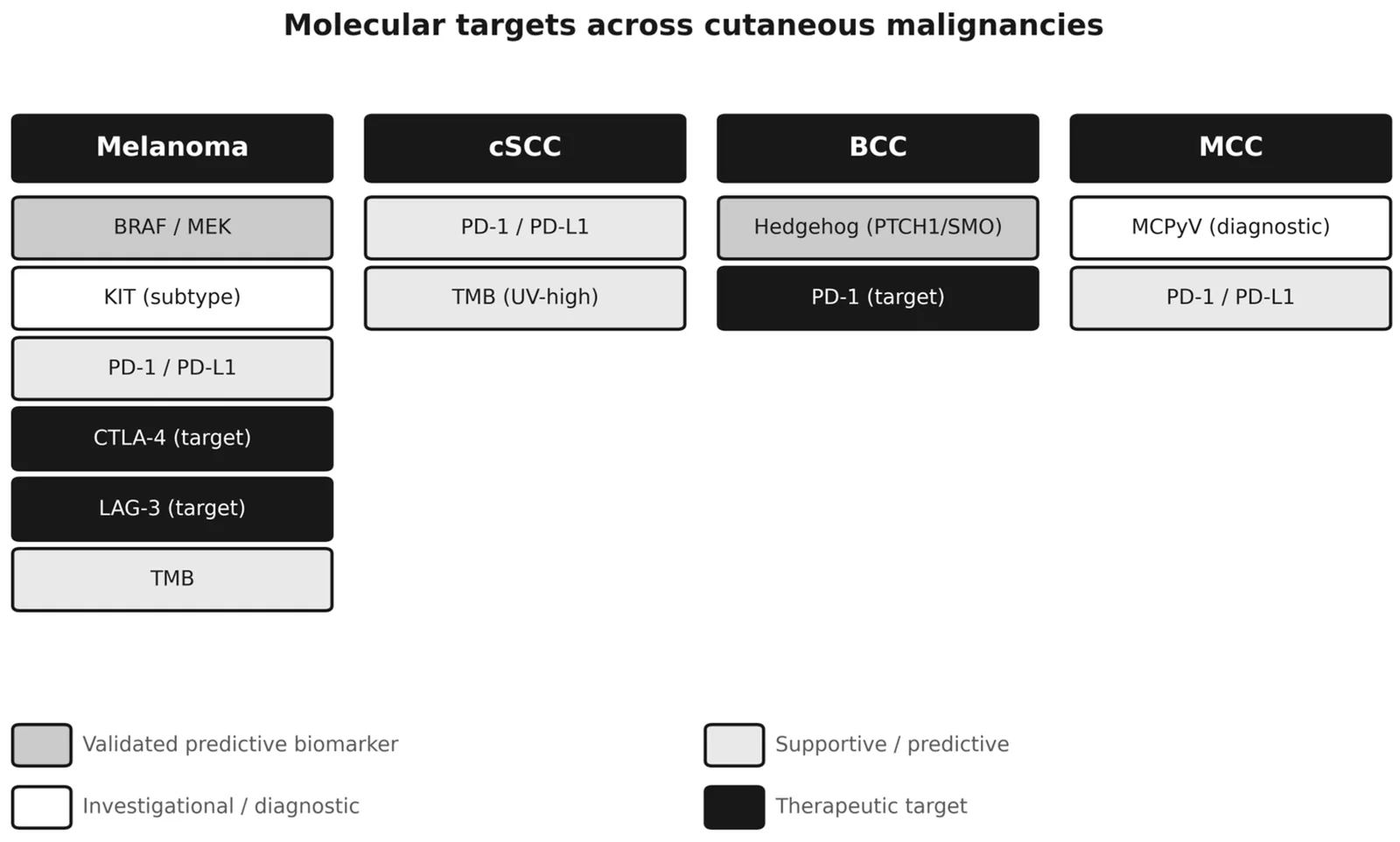
Principal molecular targets and biomarker categories across cutaneous malignancies. BRAF V600 is a validated treatment-selecting biomarker; PTCH1/SMO alterations are mechanistically relevant to Hedgehog-pathway inhibition; PD-L1, tumour mutational burden and circulating tumour DNA are context-dependent supportive or investigational markers; Merkel-cell polyomavirus has diagnostic/biological and stage-dependent prognostic relevance; and CTLA-4, LAG-3 and PD-1 are therapeutic targets rather than validated predictive biomarkers. In the European Union, tumour-cell PD-L1 <1% is specifically a regulatory eligibility criterion for nivolumab plus relatlimab in advanced melanoma and should not be generalised to other checkpoint regimens [16,25,40,71,72]. Original figure created by the authors; no third-party figure has been reproduced or adapted.

Predictive biomarkers differ by tumour type and should be distinguished from prognostic markers, regulatory selection criteria and investigational assays. BRAF V600 is a validated predictive biomarker because it determines eligibility for BRAF/MEK-targeted therapy in melanoma [1,30,31,32,33,34]. Current ESMO guidance recommends actionable mutation testing in resectable or unresectable stage III–IV melanoma and consideration in high-risk stage IIB/IIC disease; BRAF V600 testing is mandatory, while NRAS and c-KIT testing can be offered, and NGS can be used in unresectable melanoma [25]. In clinical practice, broader NGS panels can identify NRAS, KIT, NF1, TERT and co-occurring alterations that refine molecular subtype, prognosis, trial eligibility and occasionally therapeutic options beyond BRAF alone [71]. The clinical actionability of these findings is heterogeneous, however, and sequencing should not be equated with proven treatment benefit for every detected alteration. PD-L1 requires similarly careful interpretation: it is not a universally validated predictive biomarker in melanoma, but tumour-cell PD-L1 <1% is a current European regulatory selection criterion for nivolumab plus relatlimab [16]. Tumour mutational burden, circulating tumour DNA, microRNA and gene-expression profiles remain supportive or investigational in most melanoma decisions [72]. For ctDNA specifically, tumour-informed longitudinal studies demonstrate that postoperative detection is associated with higher relapse risk and may precede radiological recurrence, while a 2026 systematic review and meta-analysis confirmed strong prognostic associations but also highlighted high specificity with only modest sensitivity [73,74]. Accordingly, ctDNA is particularly promising for molecular residual disease, recurrence detection and dynamic response monitoring, yet assay standardisation, sensitivity in low-volume disease, optimal sampling intervals and prospective demonstration that ctDNA-guided intervention improves patient outcomes remain unresolved. Molecular data should therefore be integrated with stage, resectability, pathological response, disease kinetics and patient factors in multidisciplinary decision-making rather than considered in isolation. The principal molecular targets and biomarker categories are summarised in Figure 2, while their clinical statuses and roles are detailed in Table 3. Principal therapeutic agents, common trade names and biological targets relevant to cutaneous oncology are listed in Table 4.

## 11. Special Populations

Several populations require modified decision-making. In solid-organ transplant recipients, checkpoint-inhibitor therapy must be balanced against allograft rejection; registration trials largely excluded these patients, and prospective experience remains concentrated in kidney-transplant recipients [9,17,60,61]. In patients with pre-existing autoimmune disease, immunotherapy may exacerbate the underlying condition and requires individualised risk–benefit assessment and toxicity monitoring [21,22]. Older age itself is not generally an exclusion criterion in registration trials. The under-representation of older adults more often reflects comorbidity, frailty, organ dysfunction and higher ECOG/WHO performance status. Consequently, chronological age alone should not preclude treatment; decisions should instead incorporate functional reserve, geriatric vulnerability where relevant, competing comorbidity, treatment goals and the expected toxicity profile. In inherited epidermolysis bullosa—particularly recessive dystrophic epidermolysis bullosa—the risk of aggressive cSCC is high, supporting close surveillance and early assessment of suspicious lesions [77,78].

## 12. Emerging Therapies and Ongoing Trials

Several investigational strategies may further reshape practice. Talimogene laherparepvec (T-VEC) is an established intralesional oncolytic immunotherapy, but the phase III MASTERKEY-265 trial did not demonstrate significant improvement in progression-free or overall survival when T-VEC was added to pembrolizumab in advanced melanoma [79]. This negative result illustrates that biological plausibility and early-phase activity do not necessarily translate into phase III clinical benefit. Adoptive cellular therapy with tumour-infiltrating lymphocytes includes lifileucel, which received accelerated FDA approval for adults with unresectable or metastatic melanoma previously treated with a PD-1-blocking antibody and, for BRAF V600-positive disease, a BRAF inhibitor with or without a MEK inhibitor [80,81]. Personalised neoantigen therapy mRNA-4157/V940 (intismeran autogene) plus pembrolizumab improved recurrence-free outcomes in the randomised phase 2b KEYNOTE-942 study [82]. On 19 August 2026, the sponsors announced that the phase III INTerpath-001 trial met its recurrence-free-survival and distant-metastasis-free-survival endpoints; however, full peer-reviewed phase III efficacy and safety data were not available at the search cut-off and the announcement should not be interpreted as published trial evidence [83]. Bispecific antibodies and novel checkpoint targets remain earlier-stage approaches. Emerging and investigational strategies discussed in this section are summarised in Table 5.

## 13. Discussion

The contemporary management of cutaneous malignancies is defined less by the availability of individual agents than by the problem of selecting the right modality, sequence and intensity for a specific patient. Surgery remains curative for most localised keratinocyte cancers and thin node-negative melanoma, while escalation to radiotherapy or systemic therapy is driven by resectability, recurrence risk, nodal or perineural disease, expected morbidity and tumour biology [23,25,26,39]. The major recent conceptual shift is the movement of systemic therapy into the perioperative setting, where treatment can eradicate micrometastatic disease while an intact tumour remains available as an immunological substrate and—after neoadjuvant treatment—as a direct readout of pathological response [13,15]. This creates a new decision layer for multidisciplinary teams: pathological response is no longer merely prognostic but may influence whether postoperative therapy is omitted, continued or intensified.

NADINA and SWOG S1801 both favour preoperative exposure to checkpoint inhibition over an adjuvant-only strategy, but their designs answer different questions. NADINA tested neoadjuvant nivolumab plus ipilimumab with response-driven postoperative management in macroscopic stage III disease [13,14], whereas S1801 tested the timing of the same anti-PD-1 agent before and after surgery versus after surgery alone in resectable stage III–IV melanoma [15]. The practical challenge is how to act based on pathological response. Patients achieving pathological complete or major response may be candidates for treatment de-escalation within validated response-adapted pathways, reducing cumulative toxicity and cost; conversely, poor responders may warrant escalation or trial enrolment. However, response categories, pathology workflows and postoperative choices are not fully interchangeable between trials, and real-world implementation requires rapid access to surgery, standardised specimen assessment, multidisciplinary interpretation and contingency plans for progression or toxicity that may prevent surgery. Thus, the EFS advantage of neoadjuvant strategies should not be translated into a simplistic rule that every resectable patient should receive the same regimen.

Sequencing remains a central unresolved issue in advanced BRAF V600-mutated melanoma. DREAMseq supports immunotherapy-first sequencing for many patients [54], and SECOMBIT/real-world observations suggest a potential additional advantage in delaying CNS dissemination [55,56]. Nevertheless, BRAF/MEK inhibitors can produce faster tumour shrinkage and may be preferable when impending organ compromise or rapidly progressive symptomatic disease requires immediate cytoreduction. Triplet therapy does not eliminate this sequencing problem; IMspire150 led to US approval of atezolizumab plus vemurafenib and cobimetinib [52,53], but the class has not demonstrated a consistent long-term survival advantage sufficient to replace sequential strategies. Among dual immunotherapy options, 10-year CheckMate 067 data provide the most mature survival evidence for nivolumab plus ipilimumab [35], whereas nivolumab plus relatlimab offers a less toxic alternative with shorter follow-up and no randomised head-to-head comparison. The available patient-level indirect comparison is clinically informative but cannot substitute for randomisation; its published erratum should also be considered when interpreting subgroup estimates [36,36]. Accordingly, regimen choice should balance expected efficacy, toxicity, disease tempo, CNS risk and the clinical team’s capacity to manage immune-related adverse events.

Biomarker interpretation is another area in which overstatement should be avoided. BRAF V600 is a true treatment-selecting biomarker, while broader molecular profiling supplies a hierarchy of information rather than a binary therapeutic instruction. ESMO recommends actionable mutation testing in stage III–IV melanoma and allows broader NGS in advanced disease [25]; NGS can identify NRAS, KIT, NF1, TERT and co-occurring alterations that inform subtype, prognosis and trial eligibility [71]. However, the clinical actionability of many alterations remains limited, and evidence that routine broad profiling improves outcomes outside defined indications is incomplete. PD-L1 illustrates the distinction between regulatory and biological utility: tumour-cell PD-L1 <1% determines EU eligibility for nivolumab plus relatlimab [16], yet PD-L1 is not a validated universal predictor of checkpoint benefit in melanoma. Similarly, tumour mutational burden, ctDNA, microRNA and gene-expression signatures are promising but remain insufficiently standardised for routine treatment assignment [72]. Future biomarker studies should demonstrate not only analytical association but also incremental clinical utility and patient benefit.

Resistance remains a principal barrier to durable disease control and differs mechanistically among treatment classes. MAPK-pathway reactivation and bypass signalling underlie many acquired failures of BRAF/MEK inhibition [20], whereas checkpoint resistance reflects heterogeneous tumour-intrinsic and immune-microenvironment mechanisms that are not yet routinely actionable [85]. These limitations are magnified in populations under-represented or excluded from registration trials. Solid-organ transplant recipients require an explicit trade-off between cancer control and graft survival, and current prospective evidence is concentrated in kidney transplantation [61]. Older patients should not be excluded on chronological age alone; the relevant determinants are frailty, comorbidity, organ function, performance status and competing risk. For such populations, multidisciplinary risk–benefit assessment is more defensible than extrapolation from trial averages.

### Limitations of the Review

This is a narrative review and not a systematic review or quantitative synthesis. The purposive search did not retain the full record-level audit trail required for a PRISMA flow diagram, and no formal risk-of-bias instrument or certainty-grading framework was applied. The included tumours differ in biology, staging and endpoints; head-to-head comparisons between agents are often unavailable, and cross-trial comparisons are vulnerable to confounding by population, endpoint definition and follow-up. Regulatory indications also vary by jurisdiction and may change rapidly, as illustrated by the European PD-L1 restriction for nivolumab plus relatlimab. Several clinically important observations—such as anatomical-site differences in cemiplimab response and checkpoint use in transplant recipients—derive from retrospective cohorts or small prospective studies and should not be assigned the same evidentiary weight as phase III trials. Emerging phase III results disclosed only through sponsor communications were explicitly separated from peer-reviewed evidence. These constraints support cautious, guideline-anchored and multidisciplinary interpretation rather than categorical cross-trial ranking.

## 14. Future Directions

Priorities include prospectively defining the optimal sequence and duration of immunotherapy and targeted therapy; validating pathological-response-adapted perioperative algorithms; establishing whether ctDNA-guided intervention improves outcomes rather than merely predicts recurrence, despite increasingly robust prognostic evidence [73,74]; and determining when broader NGS adds actionable information beyond mandatory BRAF testing. Direct comparative evidence between dual-checkpoint regimens, prospective CNS-prevention analyses, and trials specifically designed for transplant recipients, frail older adults and other under-represented groups remain important unmet needs. For cSCC and BCC, future studies should clarify patient selection for perioperative systemic therapy, the predictive significance—if any—of anatomical site and molecular features, and strategies that maintain efficacy while minimising chronic toxicity.

## 15. Conclusions

Cutaneous oncology has entered a multimodal and increasingly precision-oriented phase, but the major clinical challenge is no longer simply access to active agents: it is selecting, sequencing and de-escalating treatment on the basis of reliable evidence. Surgery and radiotherapy remain indispensable, while perioperative immunotherapy has changed the management of selected high-risk melanoma and cSCC. BRAF V600 is an established treatment-selecting biomarker, whereas broader NGS findings, PD-L1, tumour mutational burden and ctDNA require context-specific interpretation and, in many settings, further validation. Key unresolved problems are the optimal sequencing of immunotherapy and targeted therapy, management of primary and acquired resistance, standardisation of response-driven postoperative strategies, validation of clinically useful biomarkers, and generation of prospective evidence for transplant recipients, frail older adults and other populations poorly represented in pivotal trials. Progress in these areas will determine whether precision medicine in cutaneous oncology translates from molecular description into reproducible improvement in patient outcomes.

## Figures and Tables

**Table 1:** Current treatment landscape by tumour type and clinical setting.

Tumour Type	Setting	Preferred Local Therapy	Systemic Therapy	Key Limitation
Melanoma	Resected stage IIB/IIC	Wide excision ± SLNB	Adjuvant pembrolizumab or nivolumab [27,28]	RFS/DMFS benefit; individual risk–benefit discussion and OS maturity
Melanoma	Macroscopic resectable stage III	Surgery (TLND)	Neoadjuvant ipilimumab + nivolumab; pathological-response-adapted postoperative management (NADINA) [13,14]	OS data remain immature; implementation requires expert pathology and MDT coordination
Melanoma	Advanced/metastatic	—	Anti-PD-1; nivolumab + ipilimumab; nivolumab + relatlimab (EU: PD-L1 < 1%); BRAF/MEK if V600+ [3,16,29,30,31,32,33,34,35,36,36]	Sequencing, toxicity, CNS risk; no randomised head-to-head comparison of dual-ICI regimens
cSCC	High-risk, post-op	Surgery + radiotherapy	Adjuvant cemiplimab (C-POST) [17]	No OS benefit at first analysis
cSCC	Advanced/metastatic	—	Cemiplimab; pembrolizumab; cosibelimab [9,10,37]	Solid-organ transplant recipients were excluded from pivotal trials; allograft rejection risk
BCC	Locally advanced/recurrent unresectable; metastatic (rare)	Surgery/RT if feasible	Hedgehog inhibitor; cemiplimab after HHI [7,8,38,39]	HHI cumulative toxicity; appetite/weight loss may contribute to discontinuation
MCC	Localised	Surgery + radiotherapy	Adjuvant RT; ICI under study [40]	Rarity; high recurrence
MCC	Advanced	—	Avelumab; pembrolizumab; retifanlimab [11,12,41]	Post-ICI resistance

TLND, therapeutic lymph-node dissection; RT, radiotherapy; ICI, immune checkpoint inhibitor; HHI, Hedgehog-pathway inhibitor; OS, overall survival.

**Table 2:** Pivotal trials of systemic therapy in cutaneous malignancies.

Tumour	Trial	Intervention	Key Outcome	Implication
Melanoma	NADINA (ph 3) [13]	Neoadj ipi + nivo vs. adj nivo	12 mo EFS 83.7% vs. 57.2%; HR 0.32	Neoadjuvant standard, stage III
Melanoma	SWOG S1801 (ph 2) [15]	Neoadj + adj pembrolizumab vs. adj-only pembrolizumab	2 yr EFS 72% vs. 49%	Supports perioperative timing; not a dedicated EMA neoadjuvant melanoma label
Melanoma	RELATIVITY-047 (ph 2/3) [29]	Nivo + relatlimab vs. nivo	Median PFS 10.1 vs. 4.6 mo; HR 0.75	Dual-ICI option; EU use requires tumour-cell PD-L1 < 1% [16]
Melanoma	CheckMate 067 (ph 3) [35]	Nivo + ipi vs. nivo vs. ipi	10 yr median OS 71.9 mo (nivo + ipi) vs. 36.9 mo (nivo) vs. 19.9 mo (ipi)	Longest mature survival evidence for dual PD-1/CTLA-4 blockade
cSCC	C-POST (ph 3) [17]	Adjuvant cemiplimab vs. placebo	24 mo DFS 87.1% vs. 64.1%; HR 0.32	Evidence-based adjuvant option for high-risk disease; regulatory status is jurisdiction-specific; OS immature
cSCC	EMPOWER-CSCC-1 [9]	Cemiplimab (advanced)	ORR ~47% in pivotal study; durable responses; site heterogeneity reported in real-world cohorts [51]	First-line advanced disease; higher H&N response in some cohorts is exploratory, not a validated selection factor
BCC	ERIVANCE/BOLT [7,8]	Vismodegib/sonidegib	ORR ~30–60% (locally advanced)	First-line advanced BCC
MCC	JAVELIN Merkel 200 [12]	Avelumab	High durable ORR	Preferred over chemotherapy

EFS, event-free survival; PFS, progression-free survival; DFS, disease-free survival; OS, overall survival; ORR, objective response rate; HR, hazard ratio.

**Table 3:** Predictive and emerging biomarkers.

Biomarker	Clinical Role	Status
BRAF V600	Selects BRAF/MEK inhibitor therapy (melanoma) [1,30,31,32,33,34]	Validated clinical standard
PTCH1/SMO	Hedgehog-pathway dependence and therapeutic relevance in BCC [7,8,39]	Established Hedgehog-pathway targets; mutation testing is not routinely required to select an HHI
PD-L1	Not a universal predictive biomarker; EU regulatory criterion (tumour-cell PD-L1 < 1%) for nivolumab + relatlimab [16]	Context-dependent/regulatory
Tumour mutational burden	Associated with ICI response; investigational/supportive [75,76]	Supportive/investigational
Merkel-cell polyomavirus	MCC biology/subtyping; prognostic associations strongest in initially localised disease, not established in metastatic disease [40]	Diagnostic/stage-dependent prognostic
NRAS/KIT/NF1/TERT	Molecular subtype, prognosis, trial eligibility; KIT may be actionable in selected acral/mucosal melanoma; broader profiles via NGS [25,71]	Selected/context-dependent
Circulating tumour DNA	Molecular residual disease, recurrence detection and response monitoring; prospective utility and standardisation still evolving [72,73,74]	Investigational
Comprehensive NGS profiling	Integrated assessment of co-occurring actionable and prognostic alterations beyond BRAF; useful particularly in advanced/unresectable melanoma [25,71]	Clinical tool in selected settings; actionability varies

ICI, immune checkpoint inhibitor; MCC, Merkel-cell carcinoma.

**Table 4:** Principal therapeutic agents, common trade names and biological targets relevant to cutaneous oncology.

Generic Name	Common Trade Name	Drug Class	Biological Target	Principal Cutaneous-Oncology Use
Pembrolizumab	Keytruda	Monoclonal antibody	PD-1	Melanoma; advanced cSCC
Nivolumab	Opdivo	Monoclonal antibody	PD-1	Melanoma
Ipilimumab	Yervoy	Monoclonal antibody	CTLA-4	Melanoma (usually with nivolumab)
Nivolumab + relatlimab	Opdualag	Fixed-dose dual antibody combination	PD-1 + LAG-3	First-line advanced melanoma; EU tumour-cell PD-L1 < 1%
Cemiplimab	Libtayo	Monoclonal antibody	PD-1	Advanced BCC after HHI; advanced and high-risk adjuvant cSCC
Avelumab	Bavencio	Monoclonal antibody	PD-L1	Advanced MCC
Retifanlimab	Zynyz	Monoclonal antibody	PD-1	Recurrent locally advanced or metastatic MCC; US indication
Vismodegib	Erivedge	Small-molecule inhibitor	SMO/Hedgehog pathway	Advanced BCC
Sonidegib	Odomzo	Small-molecule inhibitor	SMO/Hedgehog pathway	Locally advanced BCC
Dabrafenib + trametinib	Tafinlar + Mekinist	Small-molecule kinase inhibitors	BRAF + MEK	BRAF V600-mutated melanoma
Encorafenib + binimetinib	Braftovi + Mektovi	Small-molecule kinase inhibitors	BRAF + MEK	BRAF V600-mutated melanoma
Vemurafenib + cobimetinib	Zelboraf + Cotellic	Small-molecule kinase inhibitors	BRAF + MEK	BRAF V600-mutated melanoma

**Table 5:** Emerging and investigational strategies.

Strategy	Mechanism	Development Phase	Oncological-Safety Note
Intralesional therapy (T-VEC) [84]	Local immune activation	Approved T-VEC monotherapy; MASTERKEY-265 phase 3 combination with pembrolizumab negative for PFS/OS [79]	Do not infer benefit from early-phase combination signals
TIL therapy (lifileucel) [80]	Adoptive cellular therapy	Accelerated FDA approval for previously treated unresectable/metastatic melanoma [81]	Lymphodepletion toxicity
Neoantigen mRNA vaccine [82]	Personalised immunisation	Phase 2b peer-reviewed benefit; phase 3 INTerpath-001 positive by sponsor press release (19 August 2026), full data pending [82,83]	Peer-reviewed phase 3 efficacy/safety data not yet available
Bispecific antibodies	Dual immune engagement	Early phase	Cytokine-related toxicity

T-VEC, talimogene laherparepvec; TIL, tumour-infiltrating lymphocyte.

## Data Availability

No new data were created or analysed in this study. Data sharing is not applicable to this article.
